# Mitochondria: a breakthrough in combating rheumatoid arthritis

**DOI:** 10.3389/fmed.2024.1439182

**Published:** 2024-08-05

**Authors:** Shuang Li, Chenlu Huo, Anting Liu, Yan Zhu

**Affiliations:** ^1^Graduate School of Anhui University of Chinese Medicine, Hefei, Anhui, China; ^2^Department of Geriatrics, The Second Affiliated Hospital of Anhui University of Chinese Medicine, Hefei, Anhui, China

**Keywords:** mitochondria, mitochondrial dysfunction, rheumatoid arthritis, breakthrough point, synthesis

## Abstract

As a chronic autoimmune disease with complex aetiology, rheumatoid arthritis (RA) has been demonstrated to be associated with mitochondrial dysfunction since mitochondrial dysfunction can affect the survival, activation, and differentiation of immune and non-immune cells involved in the pathogenesis of RA. Nevertheless, the mechanism behind mitochondrial dysfunction in RA remains uncertain. Accordingly, this review addresses the possible role and mechanisms of mitochondrial dysfunction in RA and discusses the potential and challenges of mitochondria as a potential therapeutic strategy for RA, thereby providing a breakthrough point in the prevention and treatment of RA.

## 1 Introduction

Rheumatoid arthritis (RA) is a multifactorial chronic autoimmune disease of unknown aetiology, which typically involves the joints and mostly manifests as symmetric polyarthritis in the large and small joints (1). The pathological basis of RA is synovitis where synovial fibroblasts (SFs) and macrophages are the core target cells, which can lead to articular cartilage destruction and bone damage (2). Due to the complexity of the disease, RA can produce severe consequences such as joint and periarticular structure damage and systemic inflammation (3). In addition, due to its complex, chronic, and inflammatory autoimmune characteristics, RA is associated with many complications, such as cardiovascular disease, respiratory disease, and microvascular damage, which are common important risk factors for human mortality (4). The incidence of RA is on the rise owing to the impact of environmental degradation on human health and the increased average life expectancy attributed to the improvement of living standards. As reported, RA has an annual incidence of 3 cases/10,000 people and a prevalence of 1% worldwide, with a predilection for women (5). According to a study in China (6), male RA patients have more aggressive disease activity and are more susceptible to coronary artery disease. Consequently, it is urgent to seek individualised treatment options for addressing different disease responses in men and women. Drugs have historically been the mainstay of treatment for RA (7). However, the drawbacks of long-term drug use are gradually emerging, including failed personalised diagnosis and treatment, higher economic burden, increased drug resistance, and elevated side effects. Accordingly, it is necessary to elucidate the pathogenesis of RA and seek new therapeutic breakthroughs so as to develop more economical, safe, and effective treatment, reduce the physical and psychological stress of patients, improve their quality of life, and diminish the social burden.

As a fundamental organelle, mitochondria are known as the energy centre of cells, which execute and coordinate various metabolic processes within cells and meet the energy demands of cells through the oxidative phosphorylation (OXPHOS) system. In addition, mitochondria also exert an active effect in calcium-and damage-related molecular pattern signalling, amino acid and lipid metabolism, and apoptosis (8). Therefore, it is crucial for biological cells to maintain normal mitochondrial function. Mitochondrial dysfunction can contribute to mitochondrial DNA (mtDNA) mutations, increased mitochondrial reactive oxygen species (mtROS), and oxidative stress, which have been progressively underscored in the pathogenesis of RA over recent years (9). As a pivotal link in inflammation, mitochondrial dysfunction has been closely associated with the onset and progression of various types of inflammatory arthritis, including RA (10). This review summarises the role and mechanisms of mitochondrial dysfunction in RA and discusses the potential and challenges of mitochondria as a novel therapeutic strategy for RA, highlighting the potential of mitochondria as a promising breakthrough point for the prevention and treatment of RA.

## 2 Overview of mitochondrial dysfunction

**Figure 1:**
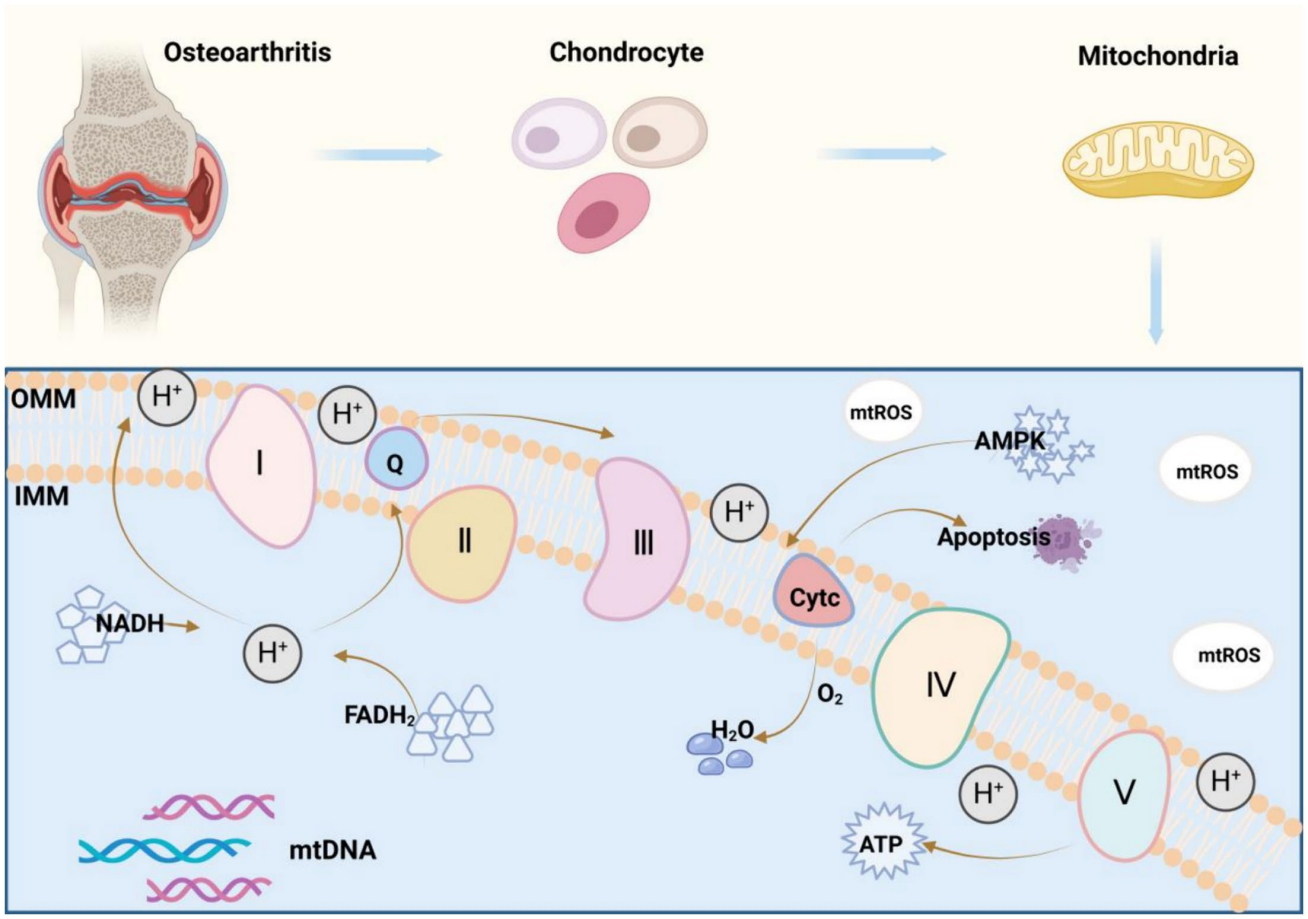
Mitochondrial oxidative phosphorylation mechanism: Mitochondria are organelles with a double-membrane structure, and their best-understood function is to produce ATP for cellular energy supply through the process of oxidative phosphorylation (OXPHOS). Nicotinamide adenine dinucleotide (NADH) and flavin adenine dinucleotide (FADH2) are the key substances in this process, which convert ADP to ATP by detaching hydrogen atoms as protons and passing them through the electron transport chain (ETC), ultimately combining them with oxygen to form water, and accompanying the release of a large amount of free energy, which allows ADP to bind to inorganic phosphoric acid. Complexes I-IV constitutes the electron transport chain (ETC), complex V is an ATP synthase, and cytochrome c and coenzyme Q also play vital roles in the mitochondrial electron transport chain, all of which are indispensable in the process of oxidative phosphorylation (OXPHOS).

Mitochondria are a double-membraned organelle with the best-known function of producing adenosine triphosphate (ATP) for cellular energy supply via OXPHOS (11). This function involves the key substances nicotinamide adenine dinucleotide (NADH) and flavin adenine dinucleotide, which generate water by allowing hydrogen atoms to detach as protons, pass along the electron transport chain (ETC), and ultimately combine with oxygen, finally resulting in the release of massive free energy and the conversion of adenosine diphosphate to ATP via inorganic phosphate (12). Complexes I-IV form the ETC, and complex V is ATP synthases (13). Cytochrome c (14) and Coenzyme Q (15) not only are vital for the mitochondrial ETC but also are indispensable in OXPHOS. Additionally, increasing studies have revealed the important mechanisms in the communication of mitochondria with other parts of cells (16), including cytochrome c release to induce cell death, 5′-adenosine monophosphate-activated protein kinase activation to control mitochondrial fission and fusion, ROS generation to activate transcription factors, and mtDNA release to activate immune responses (Figure 1). Although it is clear that impaired ATP production, mtDNA damage, and impaired inner and outer membrane transport are central events in mitochondrial dysfunction, the specific mechanisms of mitochondrial dysfunction remain unestablished (17). As mechanisms underlying mitochondrial dysfunction are extraordinarily intricate, involving multiple pathways and cytokines, in-depth research on these mechanisms can offer new ideas and approaches for the treatment and prevention of related diseases resulting from mitochondrialdysfunction.

## 3 Mechanisms of mitochondrial dysfunction in RA

### 3.1 mtDNA mutations

Human mtDNA is generally maternally inherited with a high copy number, and a typical eukaryotic cell contains hundreds to thousands of individual mtDNA molecules. New mutations may occur frequently, leading to a heterozygous state in which two or more mtDNA sequence variants (i.e., wild type and one or more mutants) coexist. Due to the high copy number of mtDNA, mutations are not scored as homozygous or heterozygous, but rather as a percentage of the total number of copies of mtDNA carrying a particular mutation, often referred to as mutation or heterozygous frequency. At low levels, mtDNA mutations have a negligible effect on the phenotype. However, an increase in mutation frequency above a critical threshold (usually 60%–80%) leads to pathogenicity and mitochondrial disease manifestations (18, 19). Since mtDNA-associated diseases were first described in the late 1980s, there have been more than 275 mutations in the mtDNA genome that cause human disease (20). It is extensively recognised that mitochondria have DNA (mtDNA) that encodes 13 key proteins for the assembly and activity of mitochondrial respiratory complexes. mtDNA is wrapped by numerous proteins to form nucleosides that are evenly distributed in the mitochondrial matrix, which is essential for mitochondrial function (21). Changes in the copy number of mtDNA (22), as a mtDNA mutation, have gradually attracted attention in recent years. A study by Picard et al. (23), which analysed the copy number of mtDNA in the blood, revealed that changes in the copy number of mtDNA elicited mitochondrial dysfunction, indicating that in the future, the quantification of the copy number of mtDNA may provide an opportunity to targeted treatment for related diseases. Lehmann Julia et al. (24) investigated plasma mtDNA levels in RA patients and found that RA patients had a high sensitivity to mtDNA copy number compared to healthy controls, and also found that plasma mtDNA concentration was significantly correlated with the Disease Activity Score-28-Erythrocyte Sedimentation Rate (DAS28-ESR), which increased with the increase of DAS28-ESR, thus it is reasonable to believe that plasma mtDNA may be an inflammatory contributor as well as a biomarker in RA. Available evidence suggests that changes in mtDNA copy number correlate with platelet abundance, and that adjusting platelet abundance and thereby affecting mtDNA copy number is of strong relevance for the development of targeted therapies for mitochondrial diseases (23). In addition, mtDNA mutations are also associated with abnormal mitochondrial ETC separation, reduced energy supply, and increased ROS production, which has been validated in the treatment of cardiovascular diseases (25). Through an independently developed low-cost single-cell mtDNA deep sequencing technology, Guo et al. (26) found that mtDNA mutations were highly pathogenic because extensive intracellular mtDNA mutations might significantly compromise mitochondrial function and promote cellular and individual senescence. Different pathogenic mtDNA mutations may affect the clinical phenotype of rheumatoid arthritis on the one hand by affecting changes in the amino acid level of the gene in question, which makes changes in the protein subunits of the gene. Some scholars have used the synoviocyte mitochondrial gene encoding NADH dehydrogenase 1 as an entry point in order to study the mutation level of synoviocyte mtDNA in rheumatoid arthritis, and have found that the number of mutations in RA synoviocyte mtDNA is approximately twice as high as that of the OA group, and that RA fibroblasts are expressed at a mutation load of 2.3 amino acid changes per thousand base pairs, whereas in tissues, there are 2.3 changes per 2.5 amino acid changes per thousand base pairs. Therefore, focusing on regulation at the amino acid level may be a direction for our future endeavours (27). On the other hand, macroscopic synovitis, vascular density, and synovial fluid cytokine TNFα levels show a positive correlation with the frequency of mutations in mtDNA, which can lead to different pathogenic mtDNA mutations, and at the same time influence the clinical phenotype of rheumatoid arthritis (28). Sliter et al. (29) observed that in a mouse model, inflammation accumulation predisposed to mtDNA mutations, and in turn, mtDNA mutations facilitated inflammation accumulation, forming a specific vicious circle between the two. Recent studies have demonstrated the inflammatory regulatory role of the mtDNA synthesis protein CMPK2, finding that CMPK2 can combine adiposity and obesity-related proteins (FTOs) in FLS to regulate synovial inflammation via the mtDNA-mediated cGAS/STING pathway, and that inhibition of the FTO—CMPK2 axis attenuates disease progression (30). Overall, mtDNA is key to proper mitochondrial function.

### 3.2 Oxidative stress

Although many chronic diseases are multifactorial in origin, they all are associated with a common process, namely oxidative stress. In the presence of exogenous stimuli (radiation, certain drugs, food, smoking, and pollution) or endogenous stimuli (mitochondrial respiratory chain), the body produces ROS, such as superoxide anion, hydroxyl radicals, and hydrogen peroxide. Specifically, superoxide anion and hydroxyl radicals, also known as free radicals, are strongly oxidative due to the existence of one or more unpaired free electrons in their outer electron orbitals, therefore easily grabbing electrons from other substances and predisposing to free radical chain reactions, which causes immense damage to DNA, proteins, and lipids (31, 32). Under physiological conditions, the antioxidant system in the body, covering antioxidant enzymes [superoxide dismutase, catalase (CAT), and glutathione (GSH) peroxidase (GPX)] and antioxidants (vitamins C and E, flavonoids, carotenoids, GSH, and micronutrients), is involved in maintaining the dynamic balance between ROS production and metabolism. Under pathological conditions, ROS overproduction or antioxidant underproduction can induce an imbalance between ROS production and metabolism, culminating in oxidative stress. Altogether, oxidative stress can be summarised as an imbalance between ROS production and the antioxidant defence system (33).

ROS production has been unravelled to play a critical role in the pathogenesis of RA by directly or indirectly inducing the participation of relevant cells in the pathogenesis of RA (34). The process of cellular oxidative phosphorylation leads to a hypoxic microenvironment, and hypoxia in the joint cavity is the condition underlying ROS accumulation and mitochondrial damage in synovial tissue (35). In turn, excess ROS cause oxidative stress, and the oxidative damage they produce is key to the induction of RA. There is evidence that tissue damage in inflammation leads to high levels of free radical production by articular chondrocytes and synovial fibroblasts (36). In turn, free radicals, especially NO and O_2_•-, inhibit the synthesis of matrix components such as proteoglycans by chondrocytes and damage the extracellular matrix through activation and up-regulation of matrix metalloproteinases, leading to cartilage damage (37). Therefore, it is reasonable to believe that ROS induce RA. For instance, Liu et al. (38) noted that the activation of nuclear receptor subfamily 1 group D member 1 (NR1D1), which participates in inflammation, lowered the expression of pro-inflammatory cytokines and matrix metalloproteinases (MMPs) and inhibited synovial proliferation by reducing ROS production, attenuating articular cartilage destruction in mice. In the study by Tang et al. (39), a nanoliposome system co-loaded with neutrophil membrane-fused leonurine (Leo; a natural anti-arthritic agent) and CAT (Leo@CAT@NM-Lipo) was constructed, which catalysed high levels of ROS into oxygen and improved the low-oxygen microenvironment at the lesion site, thereby successfully decreasing arthritis scores, ameliorating paw swelling and bone and cartilage damage, and reversing multi-organ dysfunction in adjuvant arthritis (AA) rats. Peroxisome proliferator-activated receptor-coactivator (PGC)-1 is a transcriptional coactivator that is a major regulator of mitochondrial biogenesis and function, including oxidative phosphorylation and reactive oxygen species detoxification. During inflammation, low levels of PGC-1 down-regulate the expression of mitochondrial antioxidant genes, induce oxidative stress, and promote the activation of NF-κB (40). PGC-1 binds to peroxisome proliferator-activated receptors (PPARs), which act on dystrophic muscular systems, resulting in a reduced ability of mitochondria to oxidatively phosphorylate, and an increased production of ROS (41). Activation of PGC1a is mediated by AMP activated protein kinase (AMPK), which is considered the guardian of cellular metabolism and mitochondrial homeostasis. Under low-energy conditions, AMPK phosphorylates specific enzymes and growth control nodes to increase ATP production and decrease ATP consumption (42). It has been shown that the AMPK-PGC1a axis plays an important role in joint inflammation, and the exosome miR—130b—3p regulates mitochondrial function and exacerbates cartilage damage by targeting the AMPK-PGC1a axis (43). In summary, the investigation into the specific mechanisms of oxidative stress-induced mitochondrial dysfunction may provide novel directions for the treatment of RA.

### 3.3 Abnormal energy metabolism

Cells in living organisms acquire energy from sugars, as do chondrocytes, which obtain energy through two major metabolic pathways, namely glycolysis and the Krebs cycle. Glucose molecules enter cells via cell-specific glucose transporter proteins. In the cytoplasm, one molecule of glucose can be converted to two molecules of pyruvate via glycolysis that involves a reaction catalysed by 10 enzymes, during which a small amount of ATP and the electron carrier NADH are generated, and pyruvate then enters the mitochondrial matrix and is oxidated by the pyruvate dehydrogenase system to acetyl-coenzyme A (Acetyl-CoA), which generates NADH through the eight steps of the Krebs cycle and participates in the functioning of the ETC, producing ATP and supplying energy for cellular activities (44). Here we would like to mention the key rate-limiting enzymes that promote pyruvate production during glycolysis, hexokinase-II (HK-II), phosphofructokinase-1 (PFK-1), and pyruvate kinase M2 (PKM2), the magnitude of whose activity directly affects the entire metabolic pathway. Amongst them, HK-II has been found to be specifically expressed in RA synovial lining and has a function in regulating FLS aggressiveness, and overexpression of HK-II leads to an increased ability of FLS migration and invasion (45). Meanwhile HK-II can act as a sensor of mitochondrial metabolic dysregulation to trigger mitosis, and regulating the intracellular localisation of HK-II may be a novel approach to regulate mitosis to prevent ischemic stress-induced cell death (46). Regarding PFK-1, it has been shown that pulsed PFK-1 activity induces a dominance of the dimer PKM2 and regulates cell proliferation (47). At the same time, two subunits of PFK-1, Pfk1p and Pfk2p, are involved in the regulation of the ph value of the intracellular environment as well as the concentration of glucose as a means to regulate the hydrolysis of ATP (48). Regarding PKM2, it is an important molecular determinant of metabolic adaptation in pro-inflammatory macrophages. An analysis of 1,633 and 1,603 protein spots from synovial FLS of RA patients and controls was performed and found that the expression of PKM1/M2 proteins was more than three times higher in RA FLS compared to controls. From this, we suspected that PKM2 has a role in inflammation (49). However, further studies have shown that silencing of PKM2 decreases the expression of the senescence biomarker p16, and that PKM2 is involved in cellular senescence but not in inflammation to regulate inflammation (50). Regarding pyruvate, recent studies have shown that the activator protein 1 (AP-1) transcription factor component c-Fos regulates chondrocyte proliferation and differentiation by balancing pyruvate flux between aerobic and anaerobic glycolysis (51). In a hypoxic environment, chondrocytes obtain energy highly dependent on glycolysis, which is a highly efficient pathway for cellular energy supply in the physiological state. However, the intermediate link is dysregulated in the pathological state, leading to mitochondrial dysfunction and insufficient energy supply to chondrocytes, which stimulates pathological changes (52). In summary, energy metabolism has a complex role in the RA process.

### 3.4 Hypoxaemia

Hypoxia is a feature of the synovium in RA. This phenomenon was first identified in 1970, when it was found that the oxygen tension in the synovial fluid of RA patients was lower than that of healthy controls and patients with osteoarthritis (53). Subsequently, synovial hypoxia was recognised as a potential causative factor in RA. Normally, atmospheric oxygen is extracted and delivered to the tissues via the blood. However, because of functional differences, pO_2_ is not uniform in the tissues of all parts of the body. The pO_2_ in the alveoli can be as high as 100 mmHg (16% O_2_), but in most tissues the pO_2_ drops to 40 mmHg (6% O_2_) (54). Under hypoxic conditions, cells initiate mechanisms to respond and adapt to this environment. One of the key regulators of this response is the transcription factor hypoxia-inducible factor (HIF) (55). The HIF family of proteins includes HIF-1α, HIF-2α, and HIF-3α, all of which play important roles in the pathogenesis of RA, and HIF-1α and HIF-2α are up-regulated in RA. HIF-1α plays a protective role in maintaining the dynamic balance of the articular cartilage matrix, whereas HIF-2α negatively decomposes the articular cartilage matrix. HIF-3α is a negative regulator of HIF-1α, HIF-2α and HIF-3α, and HIF-3α has not been studied much (56). HIF-1α can affect chondrocytes in a multifaceted manner in response to hypoxia. HIF-1α can play a role in glycolytic self-repair in degenerative chondrocytes through transcriptional regulation of Runx2 (57). Meanwhile, HIF-1α can inhibit chondrocyte catabolism and maintain the dynamic balance of articular cartilage matrix by regulating NF-κB signalling and limiting the expression of matrix metalloproteinase (MMP)-13 (58). HIF-2α directly induces the expression of catabolic factors in chondrocytes, and HIF-2α enhances the expression of Fas, which mediates chondrocyte apoptosis and regulates autophagy in chondrocytes (59). In addition, down-regulation of HIF-2α attenuated IL-1β-induced chondrocyte hypertrophy and cartilage degradation (60). From this, we can conclude that hypoxia is a causative agent of rheumatoid arthritis and a target for treatment.

### 3.5 Apoptosis

Mitochondria are the control centres of cellular life activities, they control apoptosis by integrating death signals through members of the Bcl-2 family and coordinating caspase activation through the release of cytochrome c (61). Accompanied by the release of cytochrome c into the cytoplasm, apoptotic protease activator-1 (Apaf-1) can respond to the release of cytochrome c and bind to it to form multimers, as well as contributing to the binding of caspase-9 to form apoptotic vesicles and induce apoptosis. Activated caspase-9 also activates other caspases, such as caspase-3 and caspase-7 (62). In addition to this, mitochondria release apoptosis-inducing factor (AIF), which is involved in the activation of caspases that induce apoptosis (63). Evidence suggests that caspase-3 inhibits mtROS production and is required for effective execution of apoptosis, whereas effector caspase-7 is required for apoptotic cell detachment (64). Recent studies have shown that caspase-3-mediated cleavage has the potential to inhibit IL-1β-mediated inflammatory responses in joints (65). FOXO1, a pro-apoptotic transcription factor in cartilage, was investigated and it was found that silencing of FOXO1 in chondrocytes resulted in a significant reduction in the mRNA levels of pro-apoptotic genes caspase-3, caspase-8, caspase-9 and TRAIL. Inhibition of caspase-8 and caspase-9 significantly reduced TNF-induced apoptosis in chondrocytes (66). Bax is a well-known pro-apoptotic protein of the Bcl-2 family and is involved in a large number of physiological and pathological processes. Bax is upregulated in cultured chondrocyte-like ATDC5 cells treated with IL-1. Bax has been identified as a direct target of miR-29a by luciferase reporter assay and immunoblotting. Targeting the pro-apoptotic protein Bax and increasing the expression level of miR-29a is a potential way to prevent the development of arthritis (67). Thus, apoptosis has an important role in RA.

## 4 Potential role of mitochondrial dysfunction in RA-related immune-inflammation

### 4.1 Mitochondrial dysfunction affects immune-inflammatory pathways in RA

#### 4.1.1 Nucleotide-binding oligomerization domain-like receptor pyrin domain containing 3 inflammasome pathway

Nucleotide-binding oligomerization domain-like receptor pyrin domain containing 3 (NLRP3) is an intracellular receptor that senses foreign pathogens and endogenous danger signals, which assembles with apoptosis-associated speck-like protein containing a caspase recruitment domain (ASC) and Caspase-1 to form polymeric proteins known as NLRP3 inflammasomes. NLRP3 inflammasomes encompass a central nucleotide-binding and oligomerisation (NACHT) domain, which contributes to self-oligomerisation and has ATPase activity. NLRP3 inflammasomes comprise a leucine-rich repeat structural domain at their C-terminus, which regulates NLRP3 inflammasome activity and detects endogenous alert proteins and microbial ligands (68). As reported, NLRP3 inflammasome activation plays a key role in defence against pathogens, and the mitochondria-related E3 ligase MARCH5 can interact with the NACHT domain of NLRP3 inflammasomes to mediate inflammasome activation (69).

In addition, when activated by pathogen-associated molecular patterns and damage-associated molecular patterns (DAMPs), the classical effector protein Caspase-1 in NLRP3 inflammasomes is cleaved to elicit the release of pro-inflammatory cytokines including interleukin (IL)-1β and IL-18, therefore participating in inflammation (70). Both IL-1β and IL-18 are pro-inflammatory cytokines in the IL-1 family. IL-1β has been identified as a clear causative factor for RA, and its excessive accumulation leads to bone and cartilage erosion (71). For example, Dong et al. (72) found that anticitrullinated protein antibody (ACPA) elevated IL-1β production in RA by activating NLRP3 inflammasomes, a crucial mechanism in the pathogenesis of RA. IL-18 dominantly regulates T helper (Th) cell responses in synovial fluids (73). In Th1 cells, IL-18, particularly in synergy with IL-12 (74), enhanced the production of interferon (IFN)-γ, which, as a regulator, acts on the overactivation-associated receptor SLAMF7 on RA macrophages to boost macrophage activation and amplify inflammation (75). In Th2 cells, IL-18 triggers host inflammation by producing cytokines such as IL-4, IL-5, and IL-13 (76). Moreover, Exconde et al. (77) observed that the non-classical effector proteins Caspase-4/-5/-11 could regulate the tetrapeptide sequence identity of IL-1β and IL-18, thereby modulating IL-1β and IL-18 recruitment and activation, which provides a basis for the development of new therapies for related diseases.

**Figure 2:**
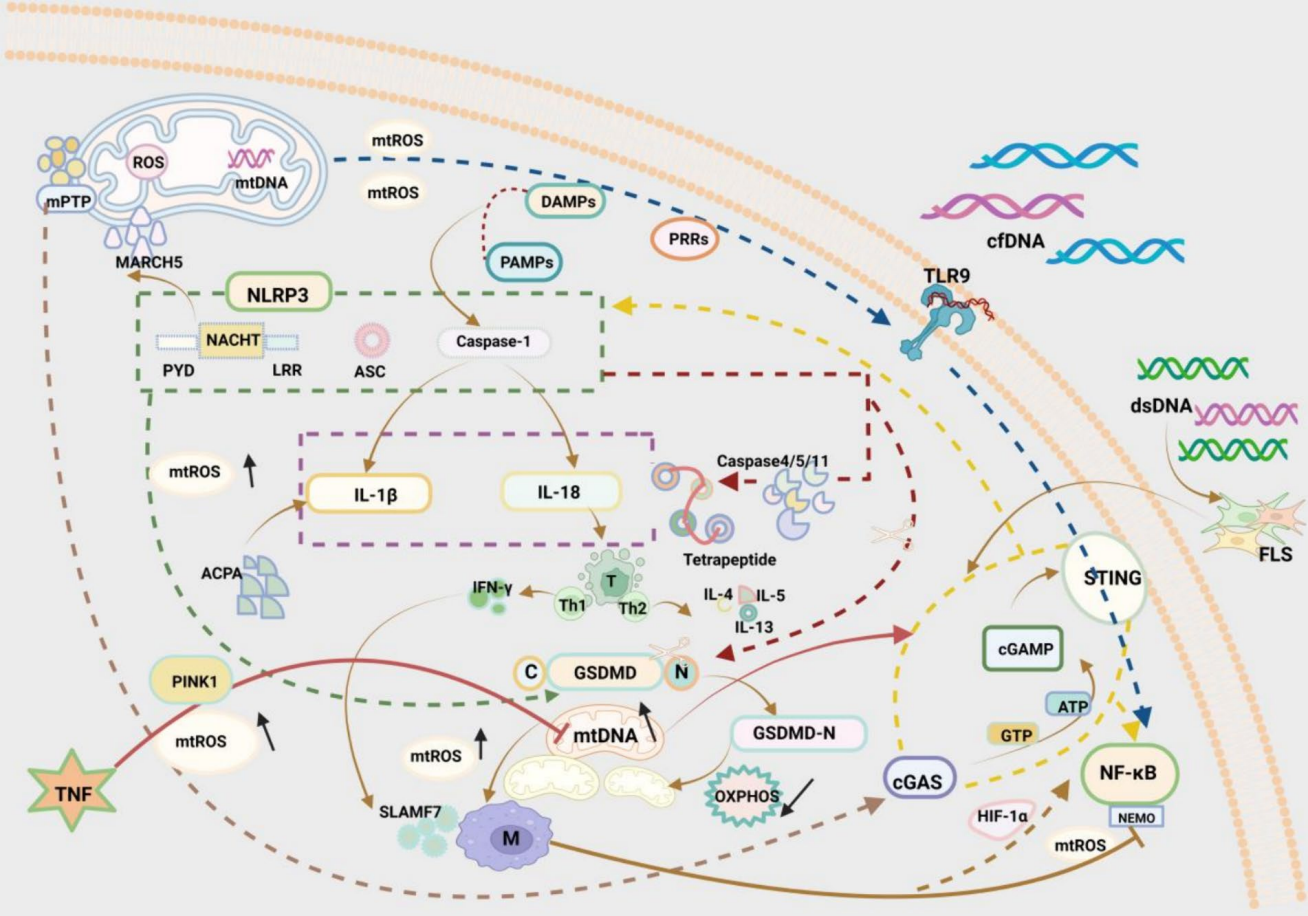
Three pathways by which mitochondrial disorders affect RA immunity: (1) *NLRP3 inflammasome pathway*: The NLRP3 inflammasome is a multimeric protein consisting of three structural domains, NACHT in the centre, PYD on the left, and LRR on the right. The mitochondrial E3 ligase MARCH5 interacts with the NACHT structural domains to regulate inflammasome activation. The classical effector protein Caspase-1 in activated NLRP3 inflammatory vesicles can promote the release of IL-1β and IL-18. IL-1β acts via ACPA in RA. IL-18 acts via regulation of T cells in RA. Gastrin D (GSDMD), a mediator of thermal apoptosis triggered by NLRP3 inflammasome, can be cleaved by caspase-1. N-terminal pore-forming GSDMD fragments (GSDMD—NT) rapidly disrupt the mitochondrial inner mitochondrial membrane (IMM) and the outer mitochondrial membrane (OMM), resulting in reduced mitochondrial autophagy and reduced oxidative phosphorylation (OXPHOS), which leads to apoptosis. The N-terminal pore-forming GSDMD fragment (GSDMD—NT) rapidly disrupts the inner mitochondrial membrane (IMM) and the outer mitochondrial membrane (OMM), leading to a reduction in the number of mitochondria, mitochondrial autophagy, and attenuation of oxidative phosphorylation (OXPHOS), which leads to apoptosis. (2) *TLR9/NF-κB pathway*: Toll-like receptor 9 (TLR9) can induce an inflammatory response through mitochondrial damage-associated molecular patterns (DAMPs) and through pattern recognition receptors (PRRs). Damaged mitochondria generate large amounts of ROS and in this way participate in the inflammatory response to RA. Cell free DNA (cfDNA) also activates TLR9, which participates in inflammation. Activated NF-κB, an evolutionarily conserved transcription factor, and hypoxia-inducible factor-1α (HIF-1α) generate a signalling cascade, which results in the production of ROS and enhances the polarisation of M1 macrophages, thus exacerbating the development of RA. In contrast, NEMO, as a fundamental regulator of NF-κB, is a regulatory subunit of NF-κB kinase (IKK) inhibitors and a potential target for inhibiting the NF-κB signalling pathway. (3) *cGAS/STING pathway*: As a cytosolic double-stranded DNA (dsDNA) sensor, cGAS recognises cytoplasmic DNA and binds to the relatively long dsDNA to change conformation and activate cGAS. activated cGAS catalyses the conversion of adenosine triphosphate (ATP) and guanosine triphosphate (GTP) to the cyclic dinucleotide (CDN) cGAMP. cGAMP stimulates STING and downstream proinflammatory factors in the inner plasma membrane and participates in immune processes. The adaptor protein STING and downstream pro-inflammatory factors are involved in the immune process. dsDNA promotes the inflammatory response through the cGAS/STING pathway in the RA FLS. Mitochondrial damage generates excessive mtROS, leading to oxidative damage to mtDNA and its release into the cytoplasm through the mitochondrial permeability transition pore (mPTP), which is accompanied by elevated levels of mtDNA and further activates the DNA sensor cGAS-STING, which activates NF-κB and NLRP3, and participates in downstream inflammatory processes.

NLRP3 inflammasomes also can trigger thermal apoptosis, which is a hot research topic. As a pivotal member of the Gasdermin (GSDM) family, GSDMD is a known inflammasome target and key pyroptotic cell death mediator (78). Caspase-1, a key effector protein of NLRP3 inflammasomes, has an essential role in RA (79). Cytoplasmic GSDMD is cleaved by Caspase-1, resulting in the release of its N-terminal domain from the autoinhibitory conformation binding to its C-terminal domain. The N-terminal domain of GSDMD (GSDMD-NT) has high affinity for acidic phospholipids, phosphatidylserine, and cardiolipin, which are rich in the inner leaflet of the plasma membrane and in the mitochondrial membrane (80). The GSDMD-NT fragment rapidly disrupts inner and outer mitochondrial membranes, contributing to a reduction in the number of mitochondria, mitochondrial autophagy, and inhibition of OXPHOS, which induces cell apoptosis (81). A prior study demonstrated that NLRP3 inflammasome activation induced the generation of mtROS, which directed the binding of GSDMD to the mitochondrial membrane, thus forming mitochondrial GSDMD pores and then causing the release of mtROS (82). mtROS release drives the oxidation of four amino acid residues of GSDMD in macrophages and markedly elevates the cleavage efficiency of Caspase-1 on GSDMD (83) (Figure 2).

NLRP3 inflammasomes are gradually being studied in the field of RA. It has been unveiled that NLRP3, Caspase-1, and cleaved GSDMD in macrophages are dramatically higher in RA than in OA. Overall, NLRP3 inflammasome inhibition emerges as a potential target for the treatment of RA (84). A prior study elucidated that the traditional Chinese medicine Qiangwu repressed mitochondrial damage, decreased the amount of mtDNA and mtROS, and inactivated NLRP3 inflammasomes, greatly reducing lipopolysaccharide-induced inflammation in AA rats (85). Another study exhibited that *acanthopanax senticosus* polysaccharides, an important active component of Acanthopanax, reprogrammed ecological dysregulation triggered by arthritis progression and enhanced γ-glutamylcysteine synthetase expression, thus suppressing the nucleation of ASC to effectively block NLRP3 inflammasome activation and attenuate inflammatory arthritis (86).

#### 4.1.2 Toll-like receptor 9/nuclear factor kappa B pathway

Toll-like receptor 9 (TLR9) is an intracellular innate immune receptor that protects the organism via diverse pattern recognition receptors (PRRs) (87). The critical involvement of TLR9 has been reported in the pathogenesis of RA. Dysfunctional mitochondria can release and misplace mitochondrial components, which serve as DAMPs and induce inflammation via PRRs, through multiple mechanisms (88). Damaged mitochondria generate large amounts of ROS whilst releasing mitochondrial DAMPs to activate PRRs, such as TLR9, thereby participating in inflammation in RA (89). TLR9 overexpression has been detected in the serum of preclinical RA subjects, which elevates inflammatory cytokine production and accelerates the progression of RA (90). To address this problem, some scholars already attempted to develop bionic nanogels as traps and cell scavengers and remove excess cells based on the high biocompatibility of bionic nanogels and found that bionic nanogels substantially depressed the TLR9 pathway and relieved arthritis symptoms in mice with collagen-induced arthritis (CIA) (91). Similarly, abnormally high cell-free DNA (cfDNA) activates TLR9 in immune cell endosomes (92), illustrating that cfDNA undoubtedly becomes a breakthrough in the treatment of RA. Methotrexate-loaded nanoparticle DNA scavengers (cNP-pp-PEG) have been developed to directly reach the core of inflammation, which not only mitigate systemic toxicity possibly induced by the strong positive charge of cationic nanoparticles (cNPs) during intravenous circulation but also enhance the therapeutic effect of cNPs (93).

The nuclear factor kappa B pathway (NF-κB) pathway is a key pathway mediating inflammation, with a pivotal role in RA pathogenesis (94). NF-κB is an evolutionarily conserved transcription factor implicated in inducible, specific, and modifiable immune responses. NF-κB also functions as a master regulator of inflammation and can be activated by multiple substances including TLRs (95). Activated NF-κB and hypoxia-inducible factor-1α (HIF-1α) produce a signalling cascade, hence generating ROS and fostering M1 macrophage polarisation to exacerbate the development of RA (96). As a basic regulator of NF-κB, NF-κB essential modulator (NEMO) is a regulatory subunit of inhibitor of NF-κB kinase and a potential target for inhibiting the NF-κB pathway. An earlier study revealed that NEMO downregulation suppressed pro-inflammatory cytokines secreted by M1 macrophages to modulate joint inflammation in CIA mice (97). Hence, NEMO deserves deep exploration (Figure 2).

#### 4.1.3 Cyclic GMP-AMP synthase/stimulator of interferon gene pathway

As a cytosolic double-stranded DNA (dsDNA) sensor, cyclic GMP-AMP synthase (cGAS) changes conformation and activates cGAS by recognising cytoplasmic DNA and binding to relatively long dsDNA. Activated cGAS catalyses the conversion of ATP and guanosine triphosphate (GTP) to cyclic guanosine monophosphate-adenosine monophosphate (a cyclic dinucleotide), which stimulates the adaptor protein stimulator of interferon gene (STING) and downstream pro-inflammatory factors on the endoplasmic membrane to participate in the immune process (98, 99). Notably, Wang et al. (100) identified cytoplasmic dsDNA accumulation as a crucial cause of fibroblast-like synoviocyte (FLS)-mediated RA synovitis, which drives inflammation primarily via the cGAS/STING pathway in RA-FLSs. Under stress conditions, mitochondrial damage results in the production of excessive mtROS, provoking oxidative damage to mtDNA and mtDNA release into the cytoplasm through the mitochondrial permeability transition pore, which further activates the DNA sensor cGAS-STING and facilitates downstream inflammatory processes (101, 102). Subsequently, cGAS-STING has been observed to show a key role in chronic inflammatory diseases by activating NF-κB and NLRP3 and then elevating the production of multiple inflammatory factors (103, 104). Tumor necrosis factor (TNF) is a core inflammatory factor in RA, which inhibits phosphatase and tensin homologue-induced kinase 1 (PINK1)-mediated mitophagy and elicits mitochondrial damage, which increases cytoplasmic mtDNA levels and activates cGAS-STING, boosting inflammation in RA (105) (Figure 2). Clearly, cGAS-STING is a potential therapeutic target for RA and deserves further investigation.

### 4.2 Mitochondrial dysfunction affects immune cells in RA

#### 4.2.1 T cells

T cells and their subsets have a crucial role in immuno-inflammation in RA (106). In the pathogenesis of RA, CD4 T cells account for a large proportion of the inflammatory cells that invade synovial tissue. In response to antigenic stimulation and cytokine signals, naïve CD4 T cells activate and differentiate into various T helper cell subsets (107). CD4 T cells can differentiate not only into IFN-γ, IL-1β, and TNF-α producing Th1 (108), but also into IL-17 producing Th17 (109). The IL-17/IL-23 axis is indispensable for Th17 maturation. Relevant studies have shown that IL-23 activates STAT3 through receptor-mediated phosphorylation, and after activation, STAT3 oligomerises and binds to the promoter region of RoRγ, thus upregulating the expression of RoRγ. STAT3 and RoRγ bind to the promoter site of the IL-17 gene, initiating the maturation and IL-17 secretion of Th17 cells. The combination of STAT3 and RoRγ binds to the IL-17 gene promoter site to initiate Th17 cell maturation and IL-17 secretion. At the same time, STAT3 also induces anti-apoptotic signals and promotes the survival of Th17 cells (110). It has been shown that Th17 is heterogeneous and is classified as ‘pathogenic’ or ‘non-pathogenic’ depending on environmental and genetic factors. ‘Pathogenic’ Th17 produces pro-inflammatory cytokines including IL-17A, IL-17F, IL-21, IL-22, and IL-26, which play a key role in the pathogenesis of chronic immune diseases (111).‘Non-pathogenic’ Th17 secretes IL-10 to negatively regulate the immune response (112). In RA, pathogenic Th17 plays an important role. In early RA, Th17 cell numbers correlate with disease activity, and nucleotide oligodomain (NOD)-like receptor 12 (NLRP12), an inhibitor, negatively regulates IL-6-induced STAT3 phosphorylation in T cells, decreasing Th17 cell differentiation and abrogating hyperinflammatory arthritis (113). In contrast, high expression of IL-17A as well as low expression of miR-26b-5p was observed in the peripheral blood of RA patients, and a negative correlation between miR-26b-5p and IL-17A was also found. miR-26b-5p can regulate the plasticity of Th17 cell differentiation in RA, prevent initial differentiation, and inhibit arthritic inflammation (114). The pathogenesis of RA is complex, and dysfunction of Tregs is one of the potential mechanisms leading to the progression of RA. Foxp3 transcription factors are spectral master regulators of Treg cell development and suppressive activity. Differentiating Tregs recognise their cognate antigens and receive T-cell receptor (TCR) signals before initiating Foxp3 transcription, which is triggered by TCR-induced transcription factors including NFAT, AP-1 and NF-κB. Once expressed, Foxp3 captures the transcriptional and epigenetic machinery induced by TCR signalling by interacting with AML1/Runx1 and NFAT. Thus, Foxp3 alters the dynamics of TCR-induced gene expression, which constitutes a major mechanism of Treg-mediated immunosuppression (115). Treg cells are the main suppressor cells for maintaining tolerance and suppressing autoantigen immunity. A related study found that Treg cell counts were expressed at a lower level in the serum of RA patients compared to OA and healthy controls, and that Treg cell counts were negatively correlated with DAS-28 scores. Improving the function of Treg cells could control joint inflammation and provide a new strategy for Treg-targeted therapy in RA (116). Recent studies have shown that an increase in the absolute and percentage of Th17 cells in the peripheral blood of patients with RA is associated with the development of atherosclerotic combined cardiovascular disease (ASCVD), and that the Th17/Treg ratio is important in the assessment of patients with RA combined with ASCVD (117). Thus, T cells and their subpopulations play a crucial role in the progression of immune inflammation in RA.

The healthy metabolism of T cells largely depends on mi tochondrial activity. In a previous study, mitochondrial fusion protein-2 (MFN2) enhanced mitochondria-endoplasmic reticulum (ER) contact and fostered inflow of mitochondrial Ca^2+^ required for efficient mitochondrial metabolism by interacting with the ER-embedded Ca-ATPase SERCA2, thereby preventing mitochondrial Ca^2+^ overaccumulation and apoptosis. Furthermore, MFN2 upregulation in CD8 T cells increases the efficacy of immunotherapy by elevating mitochondria-ER contact, which has been confirmed in anti-tumor therapy (118). A former study displayed that genetic deletion of mitochondrial pyruvate carrier (MPC) encouraged CD8 T cell differentiation towards a memory phenotype and that MPC inhibition facilitated the production of Acetyl-CoA from glutamine and fatty acid oxidation, enhancing histone acetylation and further directing T cell differentiation (119). Differentiated T cells are classified into different types, such as T follicular helper cells (Tfh) and regulatory T cells (Treg), which play an important role in the development of RA and influence the clinical phenotype of RA. Some scholars have investigated this issue, and data suggest that IL-2 inducible T-cell kinase (ITK) is crucial in the differentiation of T helper cell subpopulations, ITK activation is up-regulated in patients with RA, and ITK inhibitors alleviate arthritic inflammation by regulating the Foxo1 translocation, inhibiting the phosphorylation of PLC-γ1, an important mediator of the ITK signalling pathway, and rebalancing Th17 and Treg cells. The development of ITK inhibitors is therefore a key step in the development of the ITK pathway. Therefore, the development and use of ITK inhibitors may bring more possibilities for the treatment of RA (120).

**Figure 3:**
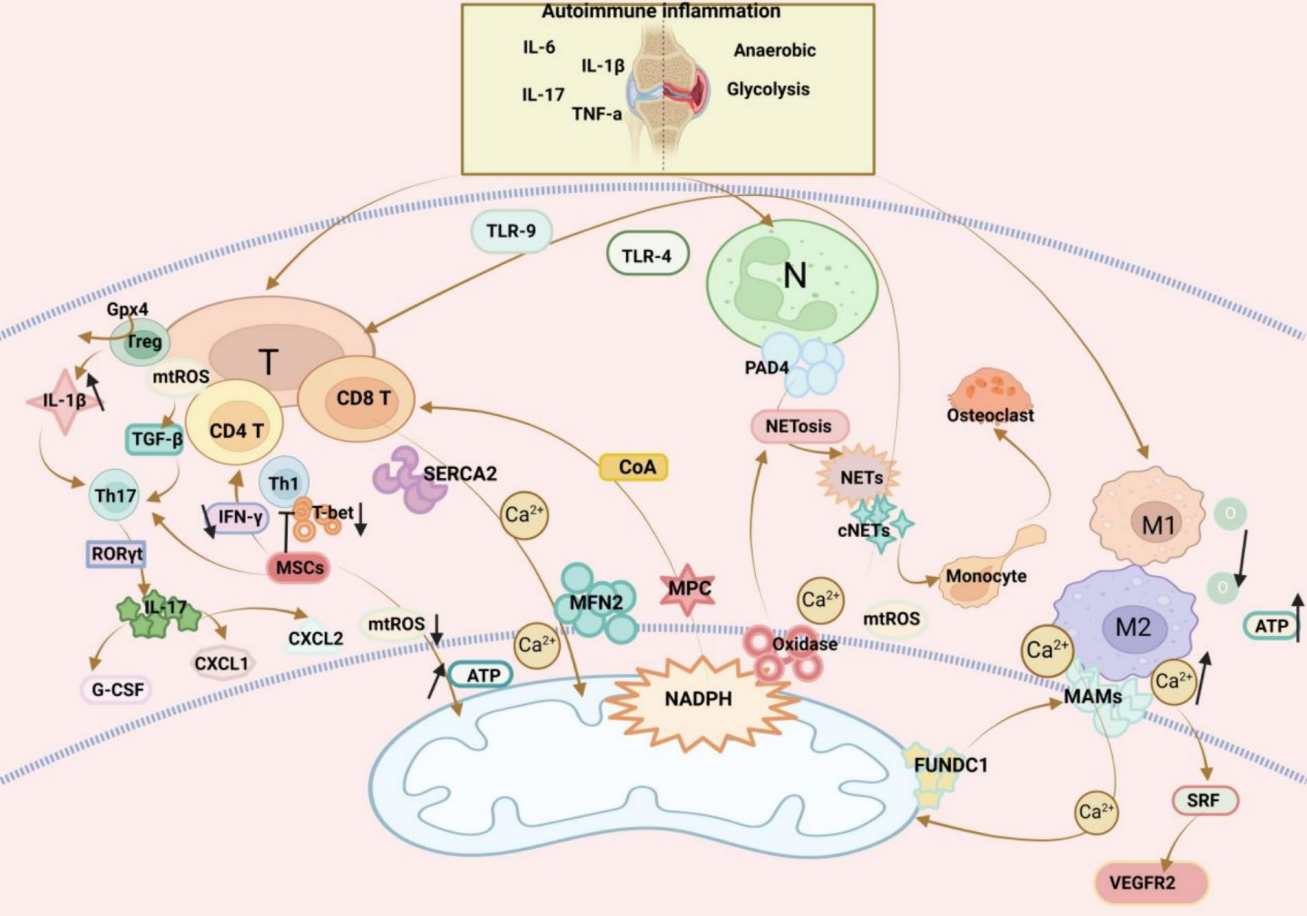
Mitochondrial dysfunction affects three immune cells in RA: (1) *T cells*: T cells and their subsets play a crucial role in the immune-inflammatory response in RA. Genetic deletion of the pyruvate carrier (MPC) drives differentiation of CD8 T cells towards a memory phenotype. Increasing MFN2 in CD8 T cells enhances mitochondrial ER contacts. ER T cells are major TNF producers. Supplementation of aspartate in T cells rescues mitochondria-directed ER membrane expansion, inhibits TNF release, and alleviates RA inflammation. Glutathione peroxidase 4 (Gpx4) prevents lipid peroxidation in Treg cells in the regulation of immune homeostasis, and Gpx4-deficient Treg cells increase IL-1β production, which promotes T helper cell 17 (Th17) responses. mtROS has a regulatory effect on T cells. mtROS in T cells activate transforming growth factor-β (TGF-β), which promotes the differentiation of Th17 cells, and then, in response to the transcription factor RORγt, produce IL-17, and finally IL-17-induced production of granulocyte colony-stimulating factor (G-CSF) and chemokines CXCL1 and CXCL2 play an inflammatory response MSCs can be transferred to activated CD4 T cells to promote T cell proliferation and reduce IFN-γ production, and inhibit Th1 responses by down-regulating the Th1 transcription factor T-bet. (2) *Macrophages*: An imbalance in the activities of pro-inflammatory M1 macrophages and anti-inflammatory M2 macrophages in RA induces synovial inflammation and autoimmunity, which can lead to joint damage. MAMs are physical contact sites between the macrophage ER and the mitochondria that mediate bidirectional communication between the two organelles. Ca^2+^ can enter mitochondria via MAMs and maintain mitochondrial homeostasis. FUN14 structural domain protein 1 (FUNDC1) is a mitochondrial outer membrane protein that mediates the formation of MAMs. Increased formation of MAMs leads to an increase in cytoplasmic Ca^2+^ levels, which promotes phosphorylation of serum response factor (SRF), enhances SRF binding to the promoter of the vascular endothelial growth factor receptor 2 (VEGFR2), leading to an increase in VEGFR2 production, and thus promotes inflammatory angiogenesis. In contrast, the formation of MAMs was reduced by silencing FUNDC1, which would inhibit angiogenesis and modulate the RA inflammatory response by decreasing VEGFR2 expression. (3) *Neutrophils*: Neutrophil infiltration of synovial joints is a feature of RA, and increased hypoxia and glycolysis in RA lead to ROS production, promoting neutrophil activation and generation. Arginine deaminase 4 (PAD4) is an enzyme that converts arginine to citrulline. PAD4 is expressed in neutrophils and activation drives the formation of neutrophil extracellular traps (NETs). After formation NETs act as activators to activate DAMP, inflammatory vesicles, antigen-presenting cells, and T cells, playing an important role in RA.

At the onset of RA, a large number of inflammatory factors such as TNF-α, IL-6, IL-17, and IL-1β accumulate in the joints, causing inflammatory storms (121). A study demonstrated that T cells in the joints have a unique ATPlo acetyl-CoAhi pro-inflammatory effector cell metabolic signature. Here, we found that deficient mitochondrial aspartate production is a key anomaly in these autoimmune T cells. Mitochondrial aspartate deficiency disrupts the regeneration of the metabolic cofactor nicotinamide adenine dinucleotide (NAD), leading to ADP-denucleosylation of the endoplasmic reticulum (ER) sensor GRP78/BiP. As a result, ribosome-rich ER membranes are enlarged, facilitating co-translational translocation and biogenesis of the transmembrane cytokine TNF. ER-rich T cells are the major TNF producers in arthritis. Therefore, we can transfer intact mitochondria into T cells as well as supplementation with exogenous aspartic acid can rescue mitochondria-induced ER membrane expansion and inhibit TNF release and inflammation in rheumatoid tissues (122). Specific deletion of Gpx4 impairs immune homeostasis but does not have a significant impact on Treg cell survival. Deletion of Gpx4 results in excessive accumulation of lipid peroxides and leads to ferroptosis of Treg cells in response to T cell receptor (TCR)/CD28 co-stimulation. Iron metabolism, in turn, affects immune homeostasis through a variety of complex mechanisms, including promoting toxic stress, inducing inflammatory responses, and damaging immune cells, leading to the development of RA. Neutralisation of lipid peroxides and blockade of iron supply rescued iron precipitation in Gpx4-deficient Treg cells. In addition, Gpx4-deficient Treg cells increase mitochondrial superoxide production and interleukin-1β (IL-1β) production, which promotes T helper cell 17 (Th17) responses and affects immune homeostasis (123, 124). mtROS exerts a regulatory effect on T cells. Specifically, mtROS in T cells activates transforming growth factor-β to accelerate the differentiation of Th17 cells, which then produce IL-17 in response to the transcription factor RORγt (125–127). Moreover, IL-17 provokes the production of granulocyte colony-stimulating factor (CSF) and chemokines including CXC motif chemokine ligand 1 (CXCL1) and CXCL2 that participate in inflammation as inflammatory mediators (128). Additionally, bone marrow-mesenchymal stem cell (MSC) therapy is gaining attention as a treatment option of immune and inflammatory diseases (129). In RA, higher levels of Th17 cells have higher pathogenicity (130). A prior study revealed that co-culture of Th17 cells with MSCs reduced pro-inflammatory cytokine secretion whilst promoting the spread of anti-inflammatory cells, thereby reducing the pathogenicity of Th17 cells (131). Another study reported that MSCs mediated mitochondrial translocation, increased ATP production, and lowered mtROS levels (132). Akhter et al. (133) also found that MSCs could be transferred to activated CD4 T cells to favour T cell proliferation and diminish IFN-γ production, therefore depressing Th1 responses by downregulating the Th1 transcription factor T-bet. Despite the deep understanding of MSCs, further research is warranted to validate their therapeutic role in RA (Figure 3).

#### 4.2.2 Macrophages

In RA, activity imbalance between pro-inflammatory M1 macrophages and anti-inflammatory M2 macrophages elicits inflammation and autoimmunity in the synovium, leading to joint damage (134). Mitochondria can reprogramme pro-inflammatory M1 macrophages to anti-inflammatory M2 macrophages, with a non-ideal efficiency. Therefore, a meta-defencor targeting mitochondria has been developed, which scavenges mtROS and suppresses mitochondrial nitric oxide synthase, thereby increasing mitochondrial transcription factor A expression, restoring aerobic respiration, and reprogramming mitochondrial metabolism for the conversion of M1 macrophages to M2 macrophages (135). Glycolysis and OXPHOS are amplified in macrophages due to immuno-inflammation in RA (136), which consumes large amounts of oxygen and produces more ATP, facilitating the tight contact of mitochondria with macrophage ER to form mitochondria-associated membranes (MAMs) (137). MAMs are physical contact sites between macrophage ER and mitochondria that mediate bidirectional communication between two organelles (138). Through MAMs, Ca^2+^ can enter mitochondria to maintain mitochondrial homeostasis (139). MAM formation can be modulated by FUN14 domain-containing 1 (FUNDC1), which is an outer mitochondrial membrane protein. As exhibited in a prior study, increased MAM formation elevated cytoplasmic Ca^2+^ levels, which promoted the phosphorylation of serum response factor (SRF) and its binding to the vascular endothelial growth factor receptor 2 (VEGFR2) promoter, producing VEGFR2 and thereby promoting inflammatory angiogenesis. Conversely, silencing FUNDC1 reduced MAM formation, decreasing VEGFR2 and repressing angiogenesis (140). The most distinctive features at the onset of RA are uncontrolled synovial hyperplasia and vascular opacification (141), which may be promising entry points for future research (Figure 3).

#### 4.2.3 Neutrophils

Neutrophil infiltration of synovial joints is a feature of RA. In RA, increased hypoxia and glycolysis lead to ROS production and neutrophil activation and production (142). Protein arginine deaminase 4, an enzyme converting arginine to citrulline, is expressed in neutrophils and drives neutrophil extracellular trap (NET) formation when activated (143). NETosis refers to the process of forming NETs. NETosis is induced dependent on ROS, which majorly originates from nicotinamide adenine dinucleotide phosphate oxidases, whose activation is dependent on an elevation in cytoplasmic Ca^2+^ concentration and occasionally on ROS generation in the mitochondria (144). After formation, NETs activate DAMPs, inflammasomes, antigen-presenting cells, and T cells, playing an essential role in RA (145). Carbamylation is a translational modification in NETs of RA patients, and carbamylated NETs (cNETs) guide the rapid formation of osteoclasts (OCs) from monocytes and accelerate bone erosion in RA (146). Prior research demonstrated that NETs caused nociceptive hypersensitivity via TLR4 and TLR9, an axis that can serve as a target for pain management in RA (147). Liu et al. (148) observed that tretinoin-loaded bovine serum albumin nanoparticles targeted inflamed neutrophils, elicited inflammatory neutrophil apoptosis, and inhibited NET release, improving RA treatment. Accordingly, it is worthwhile to further investigate the role of NETs in RA (Figure 3).

### 4.3 Mitochondrial dysfunction affects bone homeostasis in RA

Bone homeostasis in RA is inextricably linked to the metabolism of cartilage, and here we mention the important role of glutamine metabolism in relation to glycolytic processes. First, we found that chondrocyte properties and functions are closely related to glutamine metabolism. Glutamine is the most abundant amino acid in muscle and blood. The chondroblast transcription factor SOX9 stimulates glutamine metabolism by increasing glutamine consumption and levels of glutaminase 1 (GLS1). The action of GLS1 is critical for chondrocyte identity and function through a tripartite mechanism. First, glutamine epigenetically controls chondrogenic gene expression through glutamate dehydrogenase-dependent acetyl-CoA synthesis (required for histone acetylation). Second, transaminase-mediated aspartate synthesis supports chondrocyte proliferation and matrix synthesis. Third, glutamine-derived glutathione synthesis avoids accumulation of deleterious ROS, allowing chondrocytes to survive in vascular growth plates (149). Second, we found that chondrocyte properties and function correlate with glycolysis. Inflammatory chondrocytes showed higher basal extracellular acidification rate (ECAR) and more lactate production. [U-13C] Glucose labelling showed less glucose-derived carbon entering the tricarboxylic acid (TCA) cycle. On the other hand, the rate of mitochondrial respiration was significantly reduced in inflammatory chondrocytes compared to non-inflammatory chondrocytes, and these changes were accompanied by reduced cellular ATP production, decreased mitochondrial membrane potential, and disruption of mitochondrial morphology. *In vitro* studies have also shown that short-term inhibition of glycolysis suppresses the expression of matrix degeneration genes in chondrocytes and bovine cartilage explants cultured under inflammatory conditions (150). Several approaches have been proposed to correct metabolic dysregulation and restore mitochondrial function in chondrocytes to prevent or treat cartilage-related diseases. An independently controllable nanoscale plant photosynthesis system based on nano-timberoid units (NTUs) has been developed for targeting intracellular energy metabolism processes. For cross-species applications, the researchers used specific mature cell membranes [chondrocyte membranes (CM)] for camouflage encapsulation. The researchers demonstrated that these CM-NTUs enter chondrocytes through membrane fusion, avoid lysosomal degradation, and achieve rapid penetration. In addition, CM-NTUs increased intracellular ATP and NADPH levels in the presence of light, improving the metabolism of degenerating chondrocytes. They also systematically correct energy imbalance and restore cellular metabolism, thereby improving cartilage homeostasis and preventing the pathological development of osteoarthritis (151).

RA is characterised by inflammation and destruction of bones and cartilage in the affected joints. In RA, upregulation of pro-inflammatory cytokines such as TNF-α and IL-6 in the serum and joints provokes autoimmune responses, enhancing bone resorption by OCs and impairing bone formation by osteoblasts (OBs). Therefore, the basis of bone loss in RA is bone homeostasis imbalance (152, 153), in which mitochondria play an integral role. Sac/endoplasmic reticulum Ca^2+^-ATPases (SERCAs) are a family of proteins involved in calcium homeostasis in a wide range of cells and can be described as Ca^2+^ transporters. Sigma-1 receptor (Sigmar1), a specific chaperone located in mitochondria-associated endoplasmic reticulum membranes (MAMs), interacts with the SERCAs to catalyse the production of mitochondrial ATP, which carries Ca^2+^ reabsorption from the cytoplasm and carries calcium ions from the cytoplasm to the endoplasmic reticulum, a process that generates Ca oscillations and thus affects bone homeostasis in RA. Meanwhile, the researchers also found that inhibition of SERCAs impedes the fact that Sigmar1 deficiency promotes OCs production, and therefore Sigmar1 is a negative regulator of OCs production, and activation of Sigmar1 reduces OCs production (154). Transcription activator 3 (STAT3) was identified as also being located in the mitochondria-associated endoplasmic reticulum membrane (MAM) (155). The transcriptional regulator STAT3 elicits PINK1-dependent mitophagy, which eliminates dysfunctional mitochondria, reverses the collapse of the mitochondrial membrane potential, and represses mtROS release and NLRP3 inflammasomes, whilst STAT3 downregulation protects against osteoclastogenesis (156). In addition, it was further found that STAT3 in OBS is crucial for the regulation of bone metabolism, and activation of STAT3 in OBS hinders bone formation and induces bone loss in adult mice (157). STAT3 inhibitors reversed surgery-induced H-type vascularisation of the subchondral bone, significantly reducing vessel volume and number of vessels, and subchondral bone deterioration and cartilage loss were mitigated due to reduced angiogenesis. In the United States dimemorfan as a Sigmar1 agonist has also been shown to rescue bone mass in models of bone loss (158). The bone resorption process OCs requires macrophage CSF and receptor-activated NF-κB ligand (RANKL) and consumes abundant ATP produced by glycolysis and OXPHOS. Interestingly, BMP receptor type 1A (BMPR1A) deletion in OCs facilitates bone formation by OBs (159, 160). Furthermore, Ca^2+^ concentrations affect OC differentiation. Specifically, Ca^2+^ binds to and activates calmodulin, activating NFATc1b (a master transcription factor required for OC differentiation) and promoting OC differentiation (161). During this process, Ca^2+^ oscillations occur, and Fc receptor γ (FcRγ) can modulate OC production and Ca^2+^ oscillations (162). A prior study displayed that mitochondria-derived vesicles (MDVs) were indispensable in OB differentiation and that Opa1 knockdown or Fis1 overexpression in OBs enhanced mitochondrial fragmentation and favoured MDV formation, increasing mitochondrial secretion and accelerating OB generation (163). Cytidine monophosphate kinase 2 (Cmpk2), a critical mitochondria-associated gene, elevates free mtDNA production under normal conditions, leading to the formation of inflammasome-mediated inflammatory factors. A prior study revealed that Cmpk2 inhibition increased OB differentiation by improving mitochondrial function (164).

**Figure 4:**
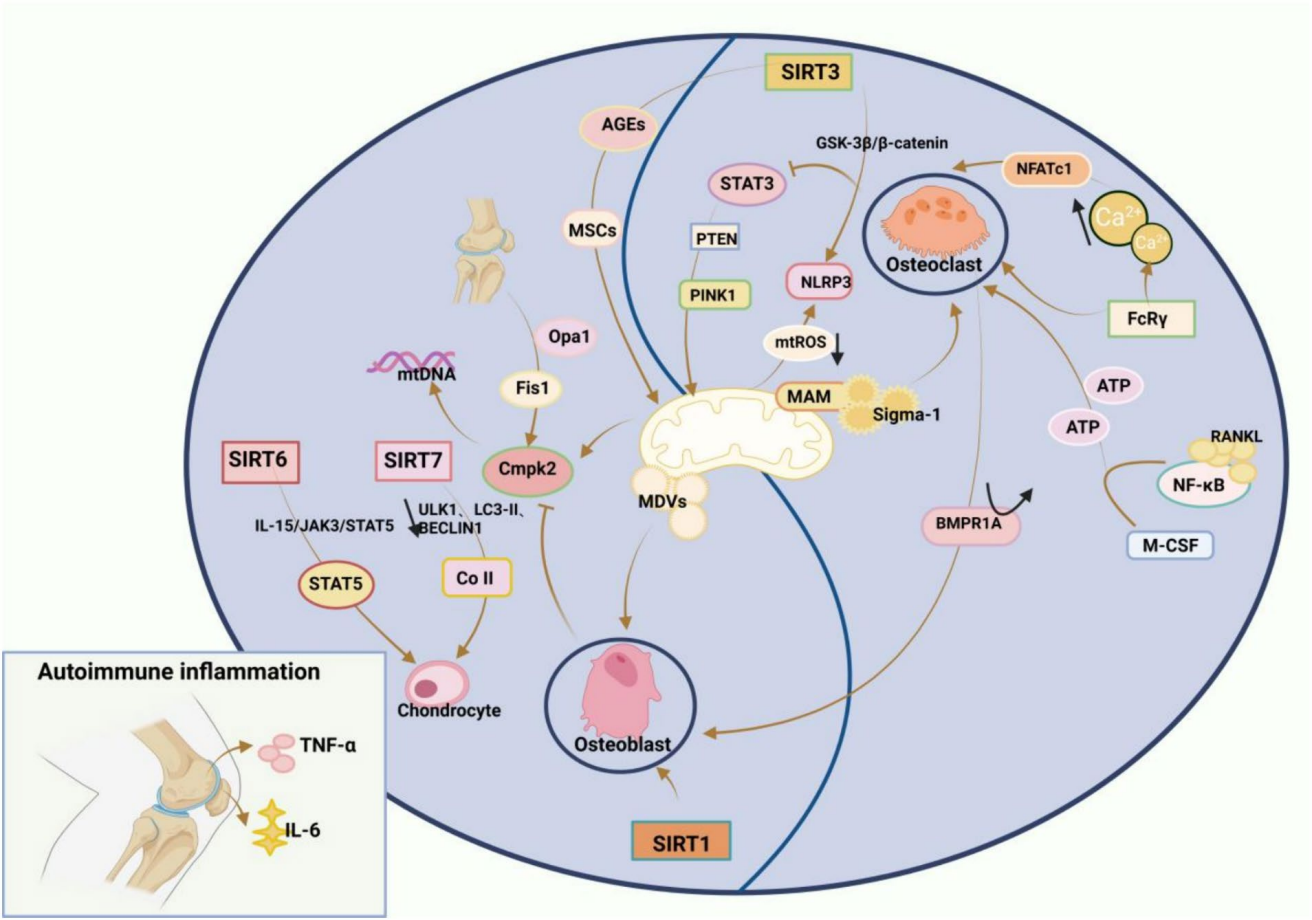
Mitochondrial disorders affect RA bone homeostasis: Increased bone resorption by osteoclasts (OCs) and impaired bone formation by osteoblasts (OBS), and an imbalance in intraosseous homeostasis underlie RA bone loss. Sigma-1 receptor (Sigmar1), a specific chaperone located in mitochondria-associated endoplasmic reticulum membranes (MAMs), is a negative regulator of OCs production, and activation of Sigmar1 reduces OCs production. The transcriptional regulator STAT3 triggers PTEN-induced kinase 1 (PINK1)-dependent mitophagy, which eliminates dysfunctional mitochondria, reverses the collapse of mitochondrial membrane potential, inhibits the release of mtROS, and inactivates NLRP3 inflammasomes, and inhibition of STAT3 reduces osteoclastogenesis. The process of bone resorption by OCs is inseparable from macrophage colony-stimulating factor (M-CSF) and receptor-activated NF-κB ligand (RANKL), and BMP receptor type 1A (BMPR1A) deficiency in OCs increases bone formation in OBS. Ca concentration also affects osteoblast differentiation, where Ca binds to calmodulin and subsequently activates calmodulin, leading to the activation of NFATc1, the master transcription factor required for the differentiation of OCs, which promotes OCs differentiation. During this process, Ca oscillation occurs, and Fc receptorγ (FcRγ) can regulate OCs generation and Ca oscillation. OBS differentiation cannot be achieved without mitochondria-derived vesicles (MDVs), and it is possible to enhance mitochondrial fragmentation by knocking down Opa1 or overexpression of Fis1 in osteoblasts, which promotes the formation of MDVs, thereby increasing mitochondrial secretion and accelerating OBS generation. Cytidine monophosphate kinase 2 (Cmpk2) is an important mitochondria-associated gene, which normally promotes the production of free mtDNA, leading to the formation of inflammasome-mediated inflammatory factors, and inhibition of Cmpk2 expression improves mitochondrial function and promotes OBS differentiation. In addition, the Sirtuin family, which is a transcriptional regulator of nuclear silencing information of anti-ageing molecules, is gaining attention in bone homeostasis. In the Sirtuin family, which consists of seven Sirtuins (SIRT1-7), SIRT1-SIRT6 play a key role in maintaining mitochondrial quality control, and SIRT1, SIRT3, SIRT6 and SIRT7 can directly participate in normal bone development and bone homeostasis through the regulation of osteoblasts.

The Sirtuin (SIRT) family is a transcriptional regulator of the nuclear silencing message of anti-ageing molecules and consists of seven SIRTs (SIRT1–7), which is gaining attention because of its role in bone homeostasis. In the Sirtuin family, SIRT1–6 are responsible for maintaining mitochondrial quality control. SIRT1, SIRT3, SIRT6, and SIRT7 are directly involved in normal bone development and homeostasis by regulating OBs (165). A previous study elaborated that SIRT1 overexpression attenuated DNA damage and mitochondrial dysfunction in OBs, diminished OB senescence, and maintained bone homeostasis (166). SIRT3 controls osteolysis by regulating NLRP3 inflammasomes via the GSK-3β/β-catenin pathway (167). Likewise, SIRT3 acts as a core regulatory enzyme of mitochondrial metabolism to mediate mitophagy and suppress the accumulation of advanced glycation end-products, thereby delaying bone marrow-MSC senescence and maintaining bone homeostasis (168). SIRT6 directly interacts with and deacetylates STAT5, inhibiting IL-15/JAK3-induced translocation of STAT5 from the cytoplasm to the nucleus and inactivating IL-15/JAK3/STAT5 signalling, which depresses chondrocyte senescence (169). Despite few studies on SIRT7, SIRT7 deficiency has been revealed to accelerate collagen II catabolism and lower ULK1, LC3-II, and BECLIN1 expression, repressing autophagy and affecting chondrocyte metabolism. Additionally, SIRT7 upregulation activates autophagy and protects chondrocytes from degeneration (170). Combined with our previous findings, it can be concluded that bone homeostasis affects bone metabolism and is influenced by mitochondrial dysfunction and that the role of the Sirtuin family in bone homeostasis is progressively being appreciated. Hence, bone homeostasis balance may be another therapeutic target for RA (Figure 4).

## 5 Summary and outlook

Recent research has provided new insights into the development of RA. The impact of mitochondria as a signalling centre on RA is multifaceted. For example, mitochondrial dysfunction directly or indirectly influences the cellular microenvironment, leading to damage or hyperactivation of cells involved in RA pathogenesis. According to this review, mitochondrial dysfunction in RA is attributed to three pathways, namely mtDNA mutations, oxidative stress, and abnormal energy metabolism. Meanwhile, mitochondrial dysfunction can affect RA via multiple pathways, which are also summarised in this review.

Although considerable progress has been accomplished in research on mitochondria in RA, many issues are yet to be resolved. For instance, although pathogenic mtDNA mutations impair cellular metabolism and cause cellular heterogeneity and disease, recent studies have unravelled that different mutations are associated with different clinical phenotypes and that purification choices of T cells also affect pathogenic mtDNA mutations *in vivo*, which has not been widely validated in RA and still needs to be further explored in the future (171). The concept of ‘heterogeneity’ has also been proposed in the context of mtDNA, which refers to the simultaneous presence of more than one variant in mtDNA. The heterogeneity of mtDNA exceeding the critical threshold contributes to chronic metabolic damage and mitochondrial dysfunction. Nevertheless, the critical threshold still requires further studies (18). Excessive oxidative stress in mitochondria results in the release of abundant ROS, and the strength and magnitude of ROS signalling determines the effects of its downstream factors, which can induce the development of chronic immune disorders pathologically characterised by mitochondrial damage, including RA. Accordingly, redox medicine is gradually being created, which intends to control oxidative stress in mitochondria within a reasonable range and thus provides an entry point for the prevention and control of the relevant diseases (172). Of course, research in this field remains in the pioneering phase. Therefore, additional larger studies are needed to better determine whether precise metabolic markers can be used to identify RA patients for the more precise control of mitochondrial oxidative stress. In addition, the idea of applying personalised redox therapy to increase the therapeutic efficacy of RA has recently received increasing attention from researchers, as redox processes modulate many aspects of human physiology and pathology to a certain extent. Nonetheless, many questions still need to be urgently addressed because this approach is readily influenced by multiple factors including inter-individual variability and ‘extreme’ groups. Hence, further research is merited (173).

To date, all available findings support the use of mitochondria as a breakthrough point in combating RA, and even some researchers have attempted to develop targeted therapeutic strategies. However, it is undeniable that these strategies have not been popularised due to safety, price, and other issues. Therefore, the current treatments of RA are still dominated by disease-modifying antirheumatic drugs (174). Despite great advances in research on the role of mitochondria in RA, research on their clinical significance in RA is still in the early stages. Undoubtedly, further studies are still warranted to analyse the relationship between mitochondrial dysfunction and RA, thus providing new ideas and a solid foundation for the development of more precise, efficient, and safe mitochondria-targeted therapeutic strategies for RA.

## Data availability statement

The original contributions presented in the study are included in the article/supplementary material, further inquiries can be directed to the corresponding authors.

## Author contributions

SL: Writing – original draft. CH: Writing – review & editing. AL: Writing – review & editing. YZ: Writing – review & editing.
